# Nothing but the truth: Consistency and efficiency of the list experiment method for the measurement of sensitive health behaviours

**DOI:** 10.1016/j.socscimed.2020.113326

**Published:** 2020-12

**Authors:** Aurélia Lépine, Carole Treibich, Ben D’Exelle

**Affiliations:** a University College London, Insitute for Global Health, London, UK; b Univ. Grenoble Alpes, CNRS, INRAE, Grenoble INP, GAEL, 38000, Grenoble, France; c University of East Anglia, School of International Development, Norwich, UK

**Keywords:** Senegal, Burkina Faso, Social desirability bias, Survey, List experiment, Condom use, Intimate partner violence

## Abstract

**Rationale:**

Social desirability bias, which is the tendency to under-report socially, undesirable health behaviours, significantly distorts information on sensitive behaviours, gained from self-reports and prevents accurate estimation of the prevalence of those, behaviours. We contribute to a growing body of literature that seeks to assess the performance of the list experiment method to improve estimation of these sensitive health behaviours.

**Method:**

We use a double-list experiment design in which respondents serve as the treatment group for one list and as the control group for the other list to estimate the prevalence of two sensitive health behaviours in different settings: condom use among 500 female sex workers in urban Senegal and physical intimate partner violence among 1700 partnered women in rural Burkina Faso. First, to assess whether the list experiment improves the accuracy of estimations of the prevalence of sensitive behaviours, we compare the prevalence rates estimated from self-reports with those elicited through the list experiment. Second, we test whether the prevalence rates of the sensitive behaviours obtained using the double-list design are similar, and we estimate the reduction in the standard errors obtained with this design. Finally, we compare the results obtained through another indirect elicitation method, the polling vote method.

**Results:**

We show that the list experiment method reduces misreporting by 17 percentage points for condom use and 16–20 percentage points for intimate partner violence. Exploiting the double-list experiment design, we also demonstrate that the prevalence estimates obtained through the use of the two lists are identical in the full sample and across sub-groups and that the double-list design reduces the standard errors by approximately 40% compared to the standard errors in the simple list design. Finally, we show that the list experiment method leads to a higher estimation of the prevalence of sensitive behaviours than the polling vote method.

**Conclusion:**

The study suggests that list experiments are an effective method to improve estimation of the prevalence of sensitive health behaviours.

## Highlights

• Intimate partner violence and unprotected sex are highly under-reported.
• The list experiment is internally consistent.
• The double-list experiment allows to significantly reduce standard error.
• The list experiment method leads to lower condom use than the polling vote method.

## 1 Introduction

An important source of measurement error in surveys relates to respondents' reluctance to report socially sensitive behaviour. This issue prevents researchers from obtaining valid information, which is needed to accurately estimate the prevalence of such behaviour. A commonly used method to reduce respondents’ hesitance to report sensitive behaviour is the list experiment technique. With this method, participants are randomly assigned to two groups (treatment or control) and are asked to report the number of statements that they agree with, without telling the researcher which ones. Respondents assigned to the control group are presented several non-sensitive items, while those allocated to the treatment group are presented the same statements plus the sensitive item. Comparing the average number of statements that respondents agree within the two groups provides an estimate of the prevalence of the sensitive behaviour in the treatment group.

The list experiment design has been extensively used in surveys, e.g. to elicit vote preferences (Gonzalez-Ocantos et al., 2012; Holbrook and Krosnick, 2010), views on undocumented migration (McKenzie and Siegel, 2013), prevalence of the use of micro-finance loans (Karlan and Zinman, 2012), and opinions on topics such as gay marriage (Lax et al., 2016) and racism (Krumpal, 2013). Currently, there is some debate on whether this method is reliable for obtaining accurate and efficient prevalence estimates. Several studies report challenges in terms of the consistency of the prevalence estimated using list experiment techniques (Bell and Bishai, 2019; Chuang et al., 2019). Moreover, as the list experiment adds random noise to the data, an important trade-off arises between potential bias reduction and the efficiency of the estimates. Note that when the estimated prevalence of a sensitive behaviour is higher when obtained with a list experiment than with direct reports, it suggests that the estimated prevalence rates are more accurate, but there is not enough evidence that they are free from any bias.

In our study, we contribute to the growing body of literature that seeks to assess the performance of the list experiment to improve estimates of the prevalence of sensitive behaviours by providing new evidence regarding the consistency and efficiency of this methodology. To do so, we use the double-list experiment method, which uses two different lists of non-sensitive items and where respondents on one list serve as the treatment group and on the other list as the control group (Droitcour et al., 1991).

More specifically, we will undertake the following analyses. First, we will compare the prevalence estimates obtained with the list method with those measured with a direct survey question to assess the potential of the list method to reduce under-reporting of sensitive health behaviours. Second, exploiting the double list experiment method, we will test the internal consistency of the list experiment method by comparing the estimated prevalence of the sensitive behaviour obtained from two distinct single list experiments conducted on the same sample. In previous research, we used a single list experiment to elicit the prevalence of condom use among female sex workers (FSWs) (Treibich & Lépine, 2019). We found a high over-estimation of the prevalence of condom use when the behaviour was measured with a direct question. We also showed that the factors associated with the level of condom use as estimated with the list experiment were in line with theoretical predictions. Nonetheless, we were unable to test if the prevalence of the sensitive behaviours obtained with the list experiment was still unbiased, and we found that the prevalence estimated with the list experiment had high variance, which might be problematic in the presence of small samples (Blair et al., 2018). Third, we will provide evidence on the increase in precision that can be achieved by using a double list instead of a single list experiment design and we discuss the minimum sample size required to ensure that the list experiment measure outperforms the direct report measure. Finally, for one of the studied sensitive behaviours, we will compare the results obtained with the list experiment to those obtained with another indirect elicitation method, the polling box method. In this method, all participants were provided graphical response papers to be placed in a ballot box outside the view of the interviewer.

We apply these methods to analyse two different sensitive behaviours: condom use among female sex workers (FSWs) in urban Senegal and intimate partner violence (IPV) in rural Burkina Faso. We chose these two sensitive behaviours because they represent important public health issues and are suspected in the literature of being largely misreported. Condom use is the main available means of preventing the spread of sexually transmitted infections (STIs), including HIV. Because consistent condom use is known to be the most cost-effective way to prevent HIV transmission (Cohen et al., 2004; Creese et al., 2002; Mitchell et al., 2015), it is the cornerstone of HIV prevention strategy in most countries, especially among groups at high risk of contracting HIV such as FSWs. A common feature of surveys among FSWs is a very high level of self-reported condom use; in Senegal, for example, self-reported condom use among FSWs is close to 100% (Treibich & Lépine, 2019). Yet, such safe behaviours are not consistent with the high prevalence of HIV and other STIs measured in FSWs (Dureau et al., 2016).

IPV is another key public health issue since it is estimated that 30% of women globally have experienced some form of sexual or physical violence at the hands of an intimate partner in their life (WHO, 2013). Despite this high prevalence, many studies have pointed to the possibility of under-reporting in self-reports of IPV (Chan, 2011). While in Burkina Faso, IPV is widely accepted by women under certain circumstances, with one out of three women declaring that wife-beating is justified if a woman goes out without telling her husband (Uthman et al., 2009), only a small proportion of women report experiencing IPV in the last available Demographic and Health Survey, conducted in 2010. More precisely, in a face-to-face survey, out of 10,009 women, only 0.78% reported ever experiencing any form of severe physical violence, and only 11.24% reported experiencing less severe physical violence. There is strong evidence in the literature that such prevalence estimates are likely to suffer from considerable under-reporting (Agüero & Frisancho, 2017; Bulte Lensink, 2019; Cullen, 2020; Krebs et al., 2011; Peterman et al., 2018; Traunmüller et al., 2019).

The focus on these types of sensitive behaviours also makes an important contribution to the existing literature. To date, only a limited number of studies have used list experiments to indirectly elicit the prevalence of condom use (Chong et al., 2013; Jamison et al., 2013; LaBrie and Earleywine, 2000; Treibich & Lépine, 2019) or to measure the prevalence of IPV (Agüero & Frisancho, 2017; Bulte and Lensink, 2019; Cullen, 2020; Krebs et al., 2011; Peterman et al., 2018; Traunmüller et al., 2019).

A comparison of the results for both types of sensitive behaviours allows us to test the robustness of our findings, specifically in terms of whether they apply equally to behaviour that tends to be under-reported (IPV) and over-reported (condom use). Accurate prevalence estimates of both types of sensitive behaviours are key for the design of effective and targeted policies.

The remainder of the article is organized as follows. In Section 2, we present the methodology used first by describing the double-list experiment design and its assumptions and then by explaining the consistency and efficiency tests that we implement. Section 3 presents the two data sets used in this study. The results are presented in Section 4. Finally, Section 5 discusses the results and methodological implications.

## 2 Method

After introducing the double-list experiment along with its underlying assumptions, we present the tests we will implement to verify the internal consistency of the list experiment, after which we explain how we will investigate the efficiency of the method. Finally, we compare the elicited prevalence rate with the rate obtained using another indirect method: the polling box method.

### 2.1 List experiment methodology

The list experiment or item count technique is an indirect questioning method that limits untruthful answers caused by social desirability bias, shame, or fear. The principle of the list experiment is to allocate respondents randomly to two different groups: a control group and a treatment group. Individuals allocated to the control group are presented with several non-sensitive statements. They are not asked to say whether they agree with each of the statements, but only how many of the statements they agree with. The same statements are then presented to the treatment group, with the difference that a sensitive statement is added to the series of non-sensitive statements. Assuming that the two groups have a similar opinion on the non-sensitive statements, one can deduce the share of individuals in the treatment group who agreed with the sensitive item by comparing the average number of statements with which the respondents in each group agreed (Blair and Imai, 2012; Glynn, 2013; Imai, 2011).

In our surveys, the participants in the control (treatment) group were presented with the following instructions: *“I [the interviewer] will read three (four) statements. I will then ask you how many of these statements you agree with. You should not tell me which specific statements you agree with but the number of statements that you agree with. I will give you three (four) marbles and you have to hold them in your right hand. Keep both of your hands behind your back. For each of the statements, if you agree with it, please transfer one marble from your right hand to your left hand behind you. If you do not agree with it, please do not transfer a marble. I will not be aware, and please do not inform me. At the end, I would like to know the total number of statements you agreed with. This number should correspond to the number of marbles you have in your left hand. I will now read the statements.”*

**Fig. 1:**
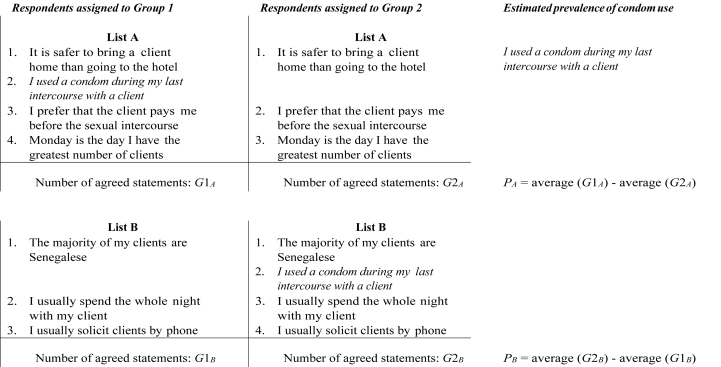
Double list experiment design - Senegalese dataset (unprotected sex) Note. Respondents assigned to group 1 serve as treated units for list A and as controls for list B while respondents assigned to group 2 serve as controls for list A and as treated for list B.

**Fig. 2:**
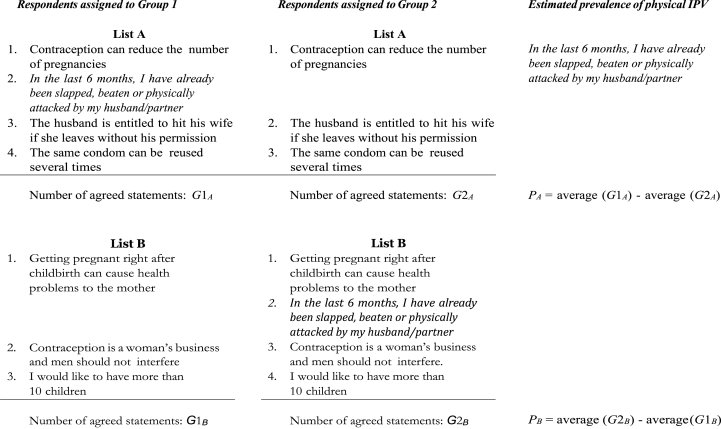
Double list experiment design - Burkina Faso dataset (intimate partner violence), Note. Respondents assigned to group 1 serve as treated units for list A and as controls for list B while respondents assigned to group 2 serve as controls for list A and as treated for list B.

We extend this methodology by using two lists instead of one, where each group served sequentially as the treated and then the control group or vice versa (Droitcour et al., 1991; Hadji et al., 2016). More precisely, the same sensitive item was used, but two different lists of non-sensitive items were presented to respondents. The ordering of the list items was identical for all respondents and everyone received list A first and list B second. As a result, some respondents first received the control list (with three non-sensitive items) and then the treatment list, while other respondents first received a treatment list (including the sensitive item) and then the control list. The statements used in the two list experiments are presented in Fig. 1, Fig. 2 for the Senegal and Burkina Faso data, respectively, along with the methodology for estimating the prevalence of the sensitive behaviour with each list.

### 2.2 List experiment assumptions

The effectiveness of the list experiment methodology is based on three assumptions: (i) the successful randomisation of the treatment, (ii) the absence of design effects, and (iii) the absence of ceiling and floor effects. More precisely, individuals allocated to each group must be similar such that on average, they agree with the same number of non-sensitive statements. Second, the addition of the sensitive item must not change the sum of affirmative answers on the control items. Finally, as pointed out by Kuklinski et al. (1997), individuals may provide untruthful answers if they no longer benefit from the privacy of their responses because they either agree or disagree with all the non-sensitive items. We refer to such effects as the ceiling and floor effects, respectively. Glynn (2013) highlights that to eliminate this problem, there should be one non-sensitive item that most participants would agree with and another non-sensitive item that most participants would disagree with. Blair and Imai (2012) also advise choosing non-sensitive items that are related to the topic of the behaviour or opinion investigated in the list experiment to avoid any suspicion on the part of respondents. The choice of the non-sensitive items is key to implementing the list experiment method successfully. Several studies (Droitcour et al., 1991; Hinsley et al., 2019; Kuklinski et al., 1997) advise that the non-sensitive items should be reasonably familiar to the respondent and sufficiently similar in nature and specificity to the sensitive item so as not to introduce bias in the answers. Hinsley et al. (2019) also mention that the non-sensitive items should not themselves be susceptible to social desirability bias.

We account for those elements in our double-list experiment design. Similar to the sensitive item, the non-sensitive items on the two lists were chosen by making sure that they referred to the sensitive behaviour of interest: sex work for the Senegalese dataset on condom use among FSWs and family planning for the Burkina Faso dataset on physical IPV. Also, the design included at least one non-sensitive item that most participants would agree with (*“I prefer that the client pays me before sexual intercourse”* on list A and *“The majority of my clients are Senegalese”* on list B for the Senegalese dataset; *“Contraception can reduce the number of pregnancies”* on list A and *“Getting pregnant right after childbirth can cause health problems for the mother”* on list B for the Burkina Faso dataset) and one non-sensitive item that most participants would disagree with (*“Monday is the day when I have the highest number of clients”* on list A and *“I usually spend the whole night with my client”* on list B; *“The same condom can be reused several times”* on list A and *“I would like to have more than 10 children”* on list B for the Burkina Faso dataset). A previous survey containing information on these statements was used to select the non-sensitive statements.

The success of the randomisation (assumption (i)) was assessed by comparing a series of individual sociodemographic characteristics among the treated and control groups.

In addition, we implemented two statistical tests (Blair and Imai, 2012) to verify whether the addition of the sensitive item modified the answers to the non-sensitive statements (assumption (ii)). More precisely, the absence of a design effect implies that:

(1) $$\mathrm{Pr}\left(Y_{i}\leq \;y\left|T_{i}=0\right)\geq \mathrm{Pr}\left(Y_{i}\leq y\right|T_{i}=1\right)\;\text{for}\;\text{all}\;y=\text{0,...,}\;3$$

(2) $$\mathrm{Pr}\left(Y_{i}\leq y\left|T_{i}=1\right)\geq \mathrm{Pr}\left(Y_{i}\leq y-1\right|T_{i}=0\right)\;\text{for}\;\text{all}\;y=1\text{,...,}4$$

where *Y*_*i*_ stands for the number of statements that the respondent agreed with and *T*_*i*_ takes the value of 1 if the respondent is allocated to the treatment group (the list including the sensitive item) and 0 otherwise.

In other words, the proportion of individuals in the control group who agree with no more than *y* statements (*y* = 0*,* 1, 2, 3) should be greater than this proportion for the treated group, and the latter proportion (for *y* = 1, 2, 3, 4) should be greater than the proportion of individuals in the control group who agree with no more than *y −* 1 statements. If this rationale is not the case, given that individuals in the treated and control groups are similar on average, it means that individuals in the treated group modified their answers to the non-sensitive items.

Finally, the potential existence of ceiling and floor effects (assumption (iii)) was investigated by looking at the share of individuals in the control group (individuals to whom only three non-sensitive items were presented) for whom *y* = 0 or *y* = 3.

### 2.3 Identification strategy

#### 2.3.1 Estimated prevalence and bias reduction

To estimate the prevalence of sensitive behaviour, we use the following regression:

(3) $$Y_{i}=\lambda +\beta^{l}T^{l}+\varepsilon_{i}$$

in which *Y*_*i*_ is the number of statements the respondent agreed with and *T*_*i*_ is a binary variable equal to one if the respondent is assigned to the treatment group and zero otherwise. The average sensitive behaviour prevalence rate is then given by *β*^*l*^ and corresponds to the average difference between the number of statements that the control group and the treatment group agreed with for each list *l* = *A, B* separately.

In a second step, we estimate the degree to which prevalence rates derived from self-reports under-estimate the frequency of the sensitive behaviour relative to estimates produced by the list experiment method. To do so, we compare the prevalence estimated with the list method with the prevalence calculated with the direct question. We use a Wald test with the null hypothesis of zero difference.

#### 2.3.2 Comparison with the polling method

**Fig. 3:**
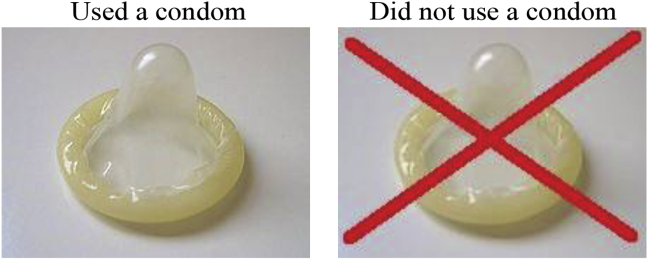
Image of condom used for the polling box.

For the Senegalese sample, we were able to compare the results of two different indirect elicitation methods. More precisely, in addition to the list experiment, the prevalence of condom use was indirectly elicited using a polling box. All FSWs were given the two pieces of paper displayed below (see Fig. 3) and were asked to put only one of them in a ballot box depending on whether they used a condom in their last sex act with a client.

Specifically, each FSW was presented with the following instruction: *“Here are two papers: One shows a condom and means that you used a male or female condom during your last commercial sex act. On the other paper, the condom is crossed out, which means that you did not use a condom during your last commercial sex act. We ask you to put in the ballot box either the paper with the picture of the condom or the one with the crossed-out condom depending on whether or not you used a condom during your last commercial sex act with a client.”*

Note that our setting did not use a perfectly confidential polling vote (see Fig. A1) because we wanted to test the feasibility of this method in an ordinary survey setting. Hence, we used the available equipment in the health facility to ensure the confidentiality of the responses.

### 2.4 Internal consistency

To test the consistency between both lists, we estimate equation (3) for each of the lists separately. We then apply a Wald test to verify whether the estimated coefficients ${\hat{\beta }}^{A}$ and ${\hat{\beta }}^{B}$ are equal. If we cannot reject the null hypothesis, we confirm the internal consistency of the sensitive behaviour prevalence obtained with the list experiment method.

As there is a possibility that the two list experiments lead to similar prevalence estimates of the sensitive behaviour “by chance”, we undertake an additional robustness test. Specifically, we test whether both lists yield similar prevalence estimates among several sub-groups, defined by characteristics that we expect to correlate with the prevalence of the sensitive behaviour. To identify relevant sub-groups, we used the literature on the determinants of condom use (Treibich & Lépine, 2019) and IPV (Angelucci, 2008; Haushofer and Shapiro, 2013; Hidrobo and Fernald, 2013; Hidrobo et al., 2016).

For this robustness test, we use equation (3) but add an interaction between the treatment dummy (*T*_*i*_) and potential factors (*S*_*i*_) influencing the occurrence of the sensitive behaviour. *β*^*l*^ reports the sensitive behaviour prevalence rate among the sub-group for which *S*_*i*_ = 0, while (*β*^*l*^ + *α*^*l*^) indicates the sensitive behaviour prevalence rate among the sub-group for which *S*_*i*_ = 1. As previously, we compare whether the two different lists provide similar prevalence rates among the sub-groups.

(4) $$Y_{i}=\lambda +\beta^{l}T_{i}+\gamma^{l}S_{i}+a^{l}T_{i}\times S_{i}+\varepsilon_{i}$$

### 2.5 Efficiency

We pool our data and add the control variable *List* (list A or list B) to equations (3) and (4) to account for our survey design with two different lists. This approach gives us the following two equations:

(5) $$Y_{i}=\lambda +\beta T_{i}+1\left(List=A\right)+\varepsilon_{i}$$

(6) $$Y_{i}=\lambda +\beta T_{i}+\gamma S_{i}+aT_{i}\times S_{i}+1\left(List=A\right)+\;\varepsilon_{i}$$

Given that each participant provided answers for lists A and B, we cluster standard errors at the individual level in regressions 5 and 6. To calculate the efficiency gains obtained by exploiting the double list, we compare the standard errors calculated from the data for only one list at a time (equations (3) and (4)) with the ones calculated from the data for both lists (i.e., equations (5) and (6)).

### 2.6 Bias-variance trade-off

The list experiment method has been shown to produce estimates closer to the actual prevalence of sensitive behaviour than those emerging from self-reports. The list method, while lowering potential bias, also adds random noise to the estimates, hence reducing their precision. Put differently, relative to the use of a list experiment, the use of a direct question may lead to higher bias in the measured prevalence, but the list experiment method could imply a higher variance in the prevalence estimate. Therefore, one may wonder under which conditions this bias-variance trade-off favours the list experiment. Using the method developed by Blair et al. (2018), we estimate the minimum sample size for which the list experiment is likely to produce more valid results than the direct question (see Appendix A3 for technical details).

We estimate the bias-variance trade-off using data collected post hoc for several reasons. First, when the bias is important and use of the direct question yields very low prevalence rates, we show with this exercise that very small samples are enough for a simple list experiment to outperform direct questioning. Second, the fact that the sample size required for the list experiment to outperform direct questioning is small (that is, the bias-variance trade-off favours the list experiment), we can also be more confident that the samples we use for our sub-group analysis are sufficiently large. Finally, even if this sample size computation is ideally conducted before data collection, it is worth checking once we have the data (and thus once we can compute the actual bias) whether our sample size is sufficiently large for the list experiment method to outperform the direct question method.

## 3 Data collection

In both studies, the questionnaire data were collected using electronic devices. We randomised the allocation of participants to the treatment or control group based on their “arrival” number. Each enumerator had to interview a specific number of participants and the arrival number refers to the order in which respondents were added to the enumerator's empty ranking sheet. Odd numbers were allocated to the treatment group of one list and the control group of the other list, whereas even numbers were assigned to the other groups. Thus, the arrival number was not manageable by the enumerator, as they did not decide who would be the next respondent to be interviewed, this ensured that the treatment assignment was orthogonal to the enumerator. Every interview lasted 1.5 hour on average and aimed to collect socio-economic, behavioural, and psychological information. After the enumerators had received enough training and practice, they could administer the double list questions in approximately 10 min, including instructions and response time. Questions were asked in Wolof in Senegal and Dioula in Burkina Faso. The translations of all questions were extensively discussed during the training of the enumerators. Ethical clearances were obtained from the London School of Hygiene & Tropical Medicine and the national ethics committee of Senegal for the survey among FSWs and from the University of East Anglia and the national ethics committee of Burkina Faso for the survey on IPV. Consent was obtained from all participants. In the rest of this section, we present more details on each data set.

### 3.1 Survey among female sex workers in Senegal

This first data set includes 495 FSWs working in Dakar, with the sample stratified by registration status (registered versus non-registered FSWs). Registered FSWs were recruited using medical records from four (out of the five) STI centres located in the suburbs of Dakar (Rufisque, Pikine, Mbao, and Sebikotane), while non-registered FSWs were recruited with the help of FSW group leaders and NGO staff. All the FSWs were asked to come to a healthcare center, where they were interviewed in dedicated private rooms. Data collection was performed in August 2017.

### 3.2 Survey among married or cohabiting women in rural Burkina Faso

Data collection was undertaken between May and July 2018 in the province of Houet, located in the southwest of Burkina Faso. In this region, we randomly selected six rural districts, and within each selected rural district, we randomly selected five villages. In each of the 30 selected villages, we conducted a census that listed all households, with information about the cohabiting or married couples in each household (some households have multiple couples). We then randomly selected 2997 households that included a married or cohabiting woman. As these data were collected to roll out a randomised controlled intervention to study couples’ fertility decisions, we also imposed the following inclusion criteria: the married or cohabiting woman (i) must be currently living with her partner/husband; (ii) must not currently be pregnant; (iii) must not be menopausal or sterilized and must not have had a hysterectomy; and (iv) must never have been told by a health worker that she has a health condition that contraindicates the use of modern contraceptives. In total, there were 1706 households with women who met these criteria. If multiple women in the same household fulfilled these conditions, we selected one woman randomly. Note that there are no missing values in the data for both samples.

## 4 Results

### 4.1 Descriptive statistics

Descriptive statistics are presented in Tables A1 and A2. In each country, we used a direct question to measure the respective sensitive behaviour. In Senegal, when asked directly, 96.77% of the interviewed FSWs declared that they had used a condom in their last paid sexual act. In Burkina Faso, 5.39% of the interviewed women reported having experienced physical IPV over the last six months. Note that the direct questions used the same wording as the sensitive item in the list experiments. In the rest of this section, we report on important socio-economic characteristics of the interviewed women in each sample.

#### 4.1.1 Senegal

The FSWs were on average 38 years old. Roughly two-thirds of the participants were divorced, and 19.80% had not yet been married. Twenty-four percent of interviewed FSWs used condoms as a contraceptive method. Their households were composed on average of seven people. In the previous two years, 6.46% had lost their mother and 9.29% their father. The average monthly income from sex work was 128,636 CFA francs (CFAF) (i.e., approximately 230 USD). Regarding their sex work activity, 40.89% (21.26%) usually worked in bars or brothels (at home). A total of 4.44% of the respondents had only occasional clients, while 35.56% had exclusively regular clients. Regarding their link with the authorities and the health system, 50.61% of respondents were registered, 36.36% of them had come to the health center in the last month, and 84.44% had had an HIV screening in the past year. Finally, 97.88% of the sample expected that they were HIV negative, and 78.98% expected that they had no STI at the time of the survey.

#### 4.1.2 Burkina Faso

Most households belonged to the Bobo (44.55%) and Mosse (26.32%) ethnic groups. The education level was low: only 24.50% and 36.34% of the women and men, respectively, had attended school, and most households were dependent on agriculture. The data show that the women tended to live with older men; on average, the women were 29 years old, and their partners were eight years older. In our sample, 25.26% of women were in polygynous unions. Most of the women in polygynous unions had one co-wife (78.90%), 16.74% had two co-wives and only 4.36% had more than three co-wives. The data show that 88.39% of couples were married. On average, the couples had been together for 10.48 years and had 3.19 children. The data also show that only 4.34% of the women could go out without the permission of their husbands.

### 4.2 List experiment assumptions

In Appendix A1, Table A1 displays the characteristics of the Senegalese FSWs in group 1 (treatment for list A and control for list B) and group 2 (control for list A and treatment for list B). Similarly, Table A2 presents a series of relevant characteristics for the Burkina Faso dataset. We observe that in both datasets, the observable characteristics are balanced between the treatment and control groups. The joint significance tests for a large share of the variables presented at the end of Tables A1 and A2 confirm the success of the randomisation (assumption (i)) for both surveys.

Based on Rows 5 and 6 of Table A3, which reports the results of the two statistical tests presented by (Blair and Imai, 2012), we can conclude that there is no design effect issue (assumption (ii)). In addition, the Bonferroni-corrected minimum *p*-values of the statistical tests indicate that we cannot reject the null hypothesis of no design effect.

In Table A3, we also note that the proportion of individuals in the control group who disagreed with all items is less than 5% (ranging from 2.4 to 4.9%, depending on the list and survey considered), which indicates a low probability that respondents in the treatment group might have felt forced to agree with the sensitive item. We also avoid the ceiling effect because the proportion of respondents in the control group who agreed with all non-sensitive items is also low (below 10%, ranging from 1.9 to 9.7%). These results support assumption (iii).

Note that these three assumptions also hold for each sub-group considered in the empirical analysis.

### 4.3 Misreporting of sensitive behaviours

Table 1 presents the prevalence of condom use in Senegal and of physical IPV in Burkina Faso as estimated using the direct survey question and each of the lists. We observe that the use of the list experiment leads to a statistically significant reduction in misreporting in both countries and that the reduction in misreporting is quite similar in both countries, ranging between 16% and 20%.

**Table 1:** Estimated prevalence and misreporting using list experiments versus direct reporting.

Senegal									
Condom use	*Ν*	Number of statements		Estimated condom use^a^	SE	95% CI	Self-reported condom use	Over- reporting	*p*-value^c^
		Treatment	Control						

*All observations*									
List A	495	2.43	1.63	0.800	0.062	[0.678; 0.922]	0.968	0.168	0.007
List В	495	2.68	1.89	0.793	0.058	[0.678; 0.908]	0.968	0.175	0.003
Lists A & B^b^	990	2.56	1.76	0.796	0.038	[0.722; 0.871]	0.968	0.171	<0.001
Burkina Faso									
IPV	*N*	Number of statements		Estimated IPV^a^	SE	95% CI	Self-reported IPV	Under reporting	*p*-value^c^
		Treatment	Control						
*All observations*									
List A	1706	1.70	1.49	0.215	0.036	[0.145; 0.285]	0.054	0.161	<0.001
List В	1706	1.70	1.44	0.261	0.035	[0.193; 0.330]	0.054	0.207	<0.001
Lists A & B^b^	3412	1.70	1.46	0.238	0.021	[0.197; 0.279]	0.054	0.184	<0.001

***Note***. N stands for number of observations, SE for standard errors, CI for confidence interval· and IPV for physical intimate partner violence.

a Estimated prevalences correspond to the β^1^ in equation (3).$Y_{i}=\lambda +\beta^{l}T_{i}+\varepsilon_{i}$

b Estimated prevalences correspond to the $\hat{\beta }$ in equation 5: Yi = *λ*+*βΤ*_*i*_ + 1 (*List = A*) +$\varepsilon_{i}$, SE are clustered at the individual level. Over-reporting and under-reporting are computed by comparing the self-reported condom use rate with the prevalence estimated with the list experiment method.

c P-values of a Wald test used to test whether the estimated prevalence differs between the direct -and indirect elicitation methods.

### 4.4 Consistency

#### 4.4.1 Internal consistency

Based on the results presented in Table 1, we can also note that the two lists used in each country provide similar prevalence estimates. In Senegal, we obtain an estimated prevalence of condom use of 80.0% with list A and 79.3% with list B. In Burkina Faso, the estimated prevalence of IPV is 21.5% with list A and 26.1% with list B. Importantly, in each country, the prevalence rates obtained with the two lists are not significantly different from each other, as demonstrated in Table 2. In the latter table, we also compare the prevalence rates for the different sub-groups. Here, we do not find any significant differences between the two lists in each country. These tests provide evidence in support of the internal consistency of the list experiment method.

**Table 2:** Comparing the two list experiments in an internal consistency test.

Senegal							
	List A^a^			List B^a^		List В-List A^b^	
	N	Prevalence	SE	Prevalence	SE	Difference	*p*-value
	(1)	(2)	(3)	(4)	(5)	(6)	(7)
All observations	495	0.800	0.062	0.793	0.058	−0.007	0.942
Price of last sex act above median (=1)	203	0.791	0.097	0.868	0.091	0.077	0.568
High HIV knowledge (=1)	410	0.791	0.067	0.806	0.063	0.015	0.865
Would be ashamed if neighbor learns about her sex work activity (=1)	426	0.855	0.066	0.790	0.063	−0.065	0.469
Registered (=1)	250	0.831	0.087	0.861	0.082	0.030	0.809
Last client was a regular client (=1)	360	0.840	0.073	0.771	0.069	−0.069	0.492
Expect to be HIV negative at the time of the survey (=1)	461	0.824	0.064	0.806	0.060	−0.018	0.832
Expect to have no STI at the time of the survey (=1)	372	0.789	0.070	0.822	0.069	0.033	0.738
Burkina Faso							
	List A^a^			List B^a^		List В - List A^b^	
	*N*	Prevalence	*SE*	Prevalence	SE	Difference	*p*-value
	(1)	(2)	(3)	(4)	(5)	(6)	(7)
All observations	1706	0.215	0.036	0.261	0.035	0.046	0.352
Polygamous marriage (=1)	436	0.179	0.071	0.211	0.069	0.032	0.725
Ever attended school (=1)	418	0.244	0.072	0.268	0.070	0.024	0.808
Did not work every month in the past year (=1)	1402	0.237	0.039	0.249	0.038	0.012	0.824
Thinks husband is entitled to beat his wife if she stands up to him (= 1)	1272	0.233	0.041	0.244	0.040	0.011	0.842
Husband ever attended school (=1)	620	0.262	0.059	0.267	0.058	0.005	0.953
Husband consumes alcohol (=1)	574	0.240	0.062	0.290	0.060	0.050	0.567
Husband does not approve contraception (=1)	647	0.193	0.058	0.188	0.057	−0.005	0.947

**Notes**. N stands for number of observations and SE for standard errors.

a Estimated prevalences reported in this table correspond to equation (3) (for all observations) and equation (4) (for subgroups).

b We compare the prevalence rates obtained with each list experiment and test the following hypothesis: *β*^*A*^ = *β*^*B*^.Differences in the number of observations for a given year is due to missing information.

#### 4.4.2 Comparison with the polling method

When using the polling box methodology, we find that the self-reported prevalence rate of condom use is higher than that obtained with the list experiment, with 88% of FSWs reporting having used a condom in their last sex act with a client. This higher rate compared to the one obtained with the list experiment methodology appears to be driven by the survey sites of Pikine and Rufisque, where the differences in the prevalence rates obtained with the list experiment and the polling box methods are greater than in the two other survey sites (see Table A4 in the Appendix).

### 4.5 Efficiency

#### 4.5.1 Reduction in standard errors with the double-list experiment

The double-list experiment design allows for a significant increase in precision, reducing the standard error by 38.7% (34.5%) for list A (list B) in terms of the measurement of protected sex and by 41.7% (40.0%) for list A (list B) in terms of the measurement of physical IPV (cf. Table 3). Similar reductions in the standard errors are obtained in the sub-group analyses.

**Table 3:** Efficiency of the double list experiment.

Senegal					
	Double list			Reduction in SE compared to	
	*N*	Prevalence	*SE*	List A	List В
	(1)	(2)	(3)	(4)	(5)
All observations	990	0.796	0.038	−0.387	−0.345
Price of last sex act above median (=1)	406	0.831	0.058	−0.402	−0.363
High HIV knowledge (=1)	820	0.799	0.042	−0.373	−0.333
Would be ashamed if neighbor learns about her sex work activity (=1)	852	0.823	0.041	−0.379	−0.349
Registered (=1)	500	0.846	0.056	−0.356	−0.309
Last client was a regular client (=1)	720	0.806	0.045	−0.384	−0.348
Expect to be HIV negative at the time of the survey (=1)	922	0.815	0.039	−0.350	−0.391
Expect to have no STI at the time of the survey (=1)	744	0.807	0.042	−0.382	−0.400
Burkina Faso					
	Double list			Reduction in SE compared to	
	*N*	Prevalence	S*E*	List A	List B
	(1)	(2)	(3)	(4)	(5)
All observations	3412	0.238	0.021	−0.417	−0.400
Polygamous marriage (=1)	872	0.195	0.039	−0.451	−0.435
Ever attended school (=1)	836	0.252	0.040	−0.444	−0.429
Did not work every month in the past year (=1)	2804	0.243	0.023	−0.410	−0.395
Thinks husband is entitled to beat his wife if she stands up to him (=1)	2544	0.238	0.025	−0.390	−0.375
Husband ever attended school (=1)	1240	0.264	0.036	−0.390	−0.379
Husband consumes alcohol (=1)	1148	0.265	0.037	−0.403	−0.383
Husband does not approve contraception (=1)	1294	0.190	0.036	−0.368	−0.379

***Note***. *N* stands for number of observations and *SE* for standard errors.

SE reduction is computed in the following way: $\frac{SE\left(\text{Double}\;\text{list}\right)-SE\left(\text{List}\;l\right)}{SE\left(\text{List}\;l\right)}\times 100$. *SE* from List A and from List B are presented in Table 1, Table 2.

#### 4.5.2 Bias-variance trade-off

We reproduce the computations presented by Blair et al. (2018) and adapt them to our case study to investigate whether our sample size is large enough to opt for prevalence elicitation through a list experiment based on the bias-variance trade-off criteria. Table 4 presents the sample size required to ensure that the list experiment method has a lower root mean square than the direct question method given the observed bias (*B*) and the estimated variance in the number of items with which the control group agrees (*V ar* (*Y*_*i*_(0))). Detailed explanations of the minimum sample size (*N*_min_) computation are presented in Appendix A3.

**Table 4:** Bias-variance trade-off and sample size.

Senegalese dataset (List A)								
Estimation of condom use	*V ar* (Y_*i*_(o))	List experiment		Direct question		*B*	N_*min*_	N_*survey*_
		π*	*SE*	*E*(*p*_*i*_)	*SE*			
All observations	0.673	0.800	0.062	0.968	0.008	0.168	94	495
Price of last sex act above median (=1)	0.665	0.791	0.097	0.966	0.013	0.175	87	203
High HIV knowledge (=1)	0.641	0.791	0.067	0.976	0.008	0.185	74	410
Would be ashamed if neighbor learns about her sex work activity (=1)	0.660	0.855	0.066	0.967	0.009	0.112	206	426
Registered (=1)	0.664	0.831	0.087	0.992	0.006	0.161	100	250
Last client was a regular client (=1)	0.670	0.840	0.073	0.961	0.010	0.121	177	360
Expect to be HIV negative at the time of the survey (=1)	0.668	0.824	0.064	0.967	0.008	0.143	128	461
Expect to have no STI at the time of the survey (=1)	0.659	0.789	0.070	0.965	0.010	0.176	84	372
Burkina dataset (List A)								
Estimation of physical IPV	*V ar* (Y_*i*_(o))	List experiment		Direct question		В	N_*min*_	N _*survey*_
		π*	SE	E (p_*i*_)	SE			
All observations	0.665	0.215	0.036	0.054	0.005	0.161	108	1706
Polygamous marriage (=1)	0.614	0.179	0.071	0.037	0.009	0.142	128	436
Ever attended school (=1)	0.666	0.244	0.072	0.062	0.012	0.182	85	418
Did not work every month in the past year (=1)	0.658	0.237	0.039	0.049	0.006	0.187	80	1402
Thinks husband is entitled to beat her wife if she stands up to him (=1)	0.650	0.233	0.041	0.055	0.006	0.178	87	1272
Husband ever attended school (=1)	0.682	0.262	0.059	0.068	0.010	0.194	77	620
Husband consumes alcohol (=1)	0.668	0.240	0.062	0.078	0.011	0.162	107	574
Husband does not approve contraception (=1)	0.698	0.193	0.058	0.048	0.013	0.156	121	647

***Note***. *N* stands for the number of observations and *SE* for standard errors. $V\;ar\left[Y_{i}\left(0\right)\right]$ is the variance in the number of statements in the control group.

$\pi^{*}$ is the true prevalence rate, which is assumed to be the result of the list experiment.

$E\left(p_{i}\right)$ is the self-declared prevalence rate in the sample. *B* is the absolute value of the difference between the self-declared and true prevalence rates.

$N_{\min}$ is the minimum sample size required so that the mean-squared error of the list experiment is lower than the MSE of the direct question.

$N_{survey}$ is the number of observations in the dataset. Let’s note $\left(x,\;y\right)=\left(N-1,\;B\right)\;\text{and}\;C=\pi^{*}\;\left(1-\pi^{*}\right)+4V\;ar\left[Y_{i}\left(0\right)\right]$ (see Appendix A3).

x is obtained by solving the following equation: $y^{2}x^{2}+\left(2\pi^{*}-1\right)yx-4V\;ar\left[Y_{i}\left(0\right)\right]x-C=0$.

‡ As an example, here we replace $\pi^{*},\;V\;ar\left[\left(Y_{i}\left(0\right)\right)\right],\;E\left(p_{i}\right)$ and *B* by their values and solve the following equation: $0.028x^{2}-2.591x-2.852=0$.

Doing so allows us to obtain *N* − 1. $N_{\min}=\left(N-1\right)+1$.

From Table 4, we can note that the required sample size is always smaller than the study sample size (*N*_*survey*_). Whereas the average biases for other attitudes or behaviours reported in the literature review by Blair et al. (2018) are approximately 5–10%, we estimate much larger biases (between 14.1% and 51.5%) in the reporting of the sensitive behaviours that we consider.

## 5 Discussion

In this study, we investigated the consistency and efficiency of the list experiment method. We demonstrated that the results of the method applied in our cases had very high internal consistency (see Table 2). We found that the use of two different lists on the same sample led to similar estimates of the prevalence of condom use (80.0% and 79.3% ) and in physical IPV (21.5% and 26.1%). We attribute this consistency to the successful fulfillment of the assumptions on which this method is based. Specifically, we demonstrated that the randomisation of the treatment assignment was successful, that there were no design effects, and that there was no indication of the presence of ceiling and floor effects, which might compromise confidentiality.

Our results also showed that imprecision arising from the noise that the list experiment method adds to the data can be substantially limited with the use of a double list instead of a simple list design, where each group serves once as the control group and once as the treatment group (Walsh and Braithwaite, 2008). In our case, the use of the double list increased the precision of our estimates by 40% (see Table 3).

Regarding bias reduction, we found that the list experiment method reduced over-reporting of condom use by 17 percentage points (see Table 1). These results are comparable to the results of other studies. LaBrie and Earleywine (2000) found that condom use was over-estimated by 11 points among college students in the United States, and Jamison et al. (2013) found it was over-estimated by 14 points among young men in Uganda, but not among young women. In addition, we find under-reporting of IPV by 16–20 percentage points. This is higher than the results of existing studies that used list experiments to estimate the prevalence of IPV. Joseph et al. (2017) found that, in India, IPV is under-reported by nine percentage points. Aguero and Frisancho (2017) find no significant difference in the prevalence rates of physical and sexual violence estimated through direct and indirect methods in Peru. Traunmüller et al. (2019) found that sexual assaults during the war in Sri Lanka were under-reported by 12 percentage points. As for Cullen (2020), she showed that IPV prevalence increased by 36% in Nigeria (26.0% vs. 19.2%) and 100% in Rwanda (20.6% vs. 9.3%) when using the list experiment instead of the direct face-to-face question.

The results of some recent studies contradict our findings. For instance, Bell and Bishai (2019) found that a list experiment led to a smaller estimate of the prevalence of sensitive behaviour than that produced by the direct question, but the authors showed that this finding was mainly due to issues with the implementation of the list experiment. They believed that participants mentally enumerated the treatment list items and the control list items in different ways. Another study by Chuang et al. (2019) concluded that the list experiment results had weak internal consistency in their case. These authors implemented several double-list experiments to measure the prevalence of sensitive sexual behaviours in Cote d’Ivoire. They found that the prevalence estimated with the two lists differed strongly for at least half of the behaviours estimated. Looking at the design of those lists, one can note that the discrepancy in the prevalence estimates could be explained by issues with several key assumptions of the list experiment methods (e.g., design effect, ceiling and floor effects). Violations of those assumptions affected the confidentiality of the responses for some lists, while confidentiality was guaranteed for others.

In sum, our study highlights the potential of list experiments to produce less biased and more efficient prevalence estimates of sensitive health behaviours than self-report-based estimates in surveys conducted in low-income countries. We are aware that without objective measures of the sensitive behaviour under study, it is impossible for even list experiments to eliminate misreporting, which is a main limitation of our study. It is difficult to think of objective measures of physical IPV or condom use in the settings that we study. In this respect, it is important to refer to other studies that have examined this issue. Haber et al. (2018) found that a list experiment had poor external validity in eliciting HIV status after the authors compared the prevalence obtained through the list experiment with that deduced through objective measures (biological markers). We hypothesise that the use of non-sensitive items unrelated to HIV status may have explained why the authors found no difference between the elicited and self-reported serostatus. Indeed, the mix of sentences such as *“I prefer bananas over grapes”* or *“I played football yesterday”* along with the sensitive item may make the sensitive item stand out too much, especially considering the stigma attached to the sensitive item under study (HIV infection). List experiment implementation guidelines stress the need to use non-sensitive items related to the sensitive item of interest (Droitcour et al., 1991; Hinsley et al., 2019; Kuklinski et al., 1997). While the above studies differ in their design, the failure of the list experiments in these studies can plausibly be attributed to violations of key assumptions of the methodology.

In addition to the impossibility of comparing the prevalence rates estimated with the list experiment to the true prevalence rates of condom use and IPV, our study has a few other limitations. First, our prevalence estimation cannot be generalised to the population of sex workers or to partnered women in Burkina Faso since our samples were not representative of those groups. Second, some of the non-sensitive statements used may be prone to social desirability bias, but given that the groups are randomised, even if the statement is considered sensitive, there is no reason to believe a priori that the misreporting would differ in the treated and control groups. Finally, we showed that the polling vote method may have failed in our study setting given the difficulty of ensuring confidentiality.

The results point to the following research needs. First, further work should examine how sensitive list experiment estimates are to violations of key assumptions of the research design. Second, further research is needed to understand why and in what context these assumptions are likely to hold in the measurement of sensitive health behaviours. Finally, more research comparing the prevalence rates estimated through multiple indirect elicitation methods and objective measures is required.

## 6 Conclusions

We tested the consistency and efficiency of the list experiment method and applied our analysis to the measurement of unprotected sex among a highly stigmatized group, FSWs in Senegal, and to the measurement of IPV among married or cohabiting women in rural Burkina Faso. We found that the method yielded results with high internal consistency. In addition, we showed that the use of a double-list experiment can significantly reduce standard errors. Finally, in the study of sensitive behaviours such as unprotected sex or physical IPV, elicitation of prevalence rates through list experiments appears to outperform the use of direct questions for small samples. In short, our results suggest that the double list experiment is a promising technique to improve the measurement of sensitive health behaviours among low-literacy populations in settings characterised by high poverty.
