# Supplementary material for: Nothing but the truth: Consistency and efficiency of the list experiment method for the measurement of sensitive health behaviours
Source: Soc Sci Med. 2020 Dec;266:113326. doi: 10.1016/j.socscimed.2020.113326 (PMC7724128; doi:10.1016/j.socscimed.2020.113326)
Supplement: Multimedia component 1 [file mmc1.pdf]

Appendices

A1 Validation of the list experiment assumptions

Table A1: Tests of randomisation and descriptive statistics - Senegal

Variables	N	Mean: All sample	Group 1	Group 2	p-value†
	N	*495*	*248*	*247*	
Socio-demographic characteristics					
Age (in years)⋆	495	38.36	38.58	38.13	0.600
Is divorced (%)⋆	495	69.09	72.58	65.59	0.093
Never married (%)⋆	495	19.80	16.94	22.67	0.110
Use contraceptive methods (%)⋆	495	68.69	70.97	66.40	0.274
Use condoms as contraceptive method (%)⋆	495	24.24	22.98	25.51	0.514
Household (HH) size⋆	495	7.01	7.07	6.95	0.805
Number of moving out in the past two years⋆	495	0.549	0.528	0.571	0.644
Mother's death after 2015 (%)⋆	495	6.46	6.05	6.88	0.707
Father's death after 2015 (%)⋆	495	9.29	8.87	9.72	0.747
HH monthly expenditures (CFAF)⋆	495	364,334	358,181	370,512	0.643
Monthly sex revenues (CFAF)⋄	489	128,636	133,608	123,643	0.329
HH received transfers in the past year (%)⋄	492	24.59	23.48	25.71	0.566
HH sent transfers in the past year (%)⋄	494	26.92	29.84	23.98	0.143
Altruism for talibe (CFAF)⋄	493	203	209	197	0.591
Altruism for sex worker (CFAF)⋄	493	97	91	103	0.535
Risk preferences in general (1 to 10)⋆	495	3.83	3.69	3.98	0.247
Risk preferences in sex (1 to 10)⋆	495	2.41	2.31	2.52	0.395
Preference for future (1 to 10)⋆	495	7.47	7.71	7.23	0.108
Trust in others⋄	488	23.36	23.87	22.86	0.792
Life satisfaction (1 to 5)⋆	495	3.15	3.15	3.15	0.994
Health status (0 to 100)⋆	495	77.42	76.73	78.11	0.531
Feeling helplessness (1 to 4)⋆	495	2.64	2.68	2.59	0.259
Fear of discrimination due to HIV (%)	443	64.33	67.57	61.09	0.155
Fear of discrimination due to sex work (%)	477	66.04	66.53	65.53	0.819
Family knows about sex work (%)⋄	483	31.06	29.51	32.64	0.459
Ashamed if neighbour learns about her activity (%)⋄	492	86.59	85.71	87.45	0.573
HIV knowledge (score 0-8)⋆	495	6.24	6.25	6.23	0.733
Sex work activity					
Work mostly in bars or brothels (%)⋄	494	40.89	40.08	41.70	0.715
Work mostly at home (%)⋄	494	21.26	20.65	21.86	0.742
Has only occasional clients (%)⋆	495	4.44	4.44	4.45	0.992
Has only regular clients (%)⋆	495	35.56	36.29	34.82	0.733
Last client was a regular client (%)⋆	495	72.73	71.77	26.32	0.634
Declared use of condom with last client (%)⋆	495	96.77	95.16	98.38	0.043
Number of clients within a week⋆	495	8.45	8.46	8.44	0.982
Price of last sex act (CFAF)⋄	494	17,134	19,609	14,658	0.133
Link with the authorities and the health system					
Legal sex worker (LSW) (%)⋄	494	50.61	51.82	49.39	0.590
Police violence in the last 12 months (%)⋆	495	5.25	5.65	4.86	0.696
Has received free condoms (%)	478	59.21	56.38	62.13	0.202
Is affiliated to a STD centre⋄	494	59.11	61.13	57.09	0.361
Came to a STD centre in the last month (%)⋆	495	36.36	37.50	35.22	0.599
Had a HIV screening in the past year (%)⋆	495	84.44	82.26	86.64	0.179
Expect to be HIV negative at the time of the survey (%)	471	97.88	98.29	97.47	0.537
Expect to have no STI at the time of the survey (%)	471	78.98	77.78	80.17	0.525
Participated in the PrEP demonstration⋆	495	19.19	17.34	21.05	0.295
**Test of joint significance**					
considering the variables indicated by ⋆:		$F(26,468) = 0.86$, p-value = 0.660			
considering the variables indicated by ⋆ and ⋄:		$F(40,432) = 0.74$, p-value = 0.876			

Notes: N stands for number of observations. Differences in the number of observations for a given year is due to missing information. † Reports the p-value of the difference of means between group 1 and group 2. Variations in the number of observations is due to missing information. HH: household; CFAF: CFA francs; HIV: human immunodeficiency virus; STD: sexually transmitted disease; STI: sexually transmitted infection; PrEP: Pre-Exposure Prohylaxis.

Table A2: Tests of randomisation and descriptive statistics - Burkina Faso

**Variables**	N	**Mean** All sample	**Mean** Group 1	**Mean** Group 2	p**-value**†
		1,706	*852*	*854*	
Socio-demographic characteristics					
Age (in years)⋆	1,706	28.72	28.71	28.73	0.913
Is married (%)⋆	1,706	88.39	88.50	88.29	0.894
Ethnic group: Bobo (%)⋆	1,706	44.55	44.13	44.96	0.729
Ethnic group: Mosse (%)⋆	1,706	26.32	26.41	26.23	0.933
Polygamous marriage (%)⋆	1,706	25.56	25.70	25.41	0.889
Years of marriage/ relationship⋄	1,698	10.48	10.42	10.54	0.686
Household size⋆	1,706	9.16	9.12	9.20	0.793
Number of children⋆	1,706	3.19	3.17	3.20	0.647
Went to school (%)⋆	1,706	24.50	23.36	25.64	0.272
Did not worked every months in the last year (%)⋆	1,706	82.18	83.45	80.91	0.171
Bargaining power and violence					
Contributes to less than half household revenues (%)⋆	1,706	84.35	84.51	84.19	0.858
Can refuse to have sex with husband (%)⋆	1,706	16.41	15.85	16.98	0.528
Can force husband to use a condom (%)⋆	1,706	14.07	13.73	14.40	0.691
Can go out without husband permission (%)⋆	1,706	4.34	4.45	4.22	0.804
Thinks a husband is entitled to hit or beat her wife if: (%)					
- she leaves the house without asking her permission⋆	1,706	47.19	46.36	48.01	0.496
- she neglects/leaves her children behind⋆	1,706	56.92	56.46	57.38	0.701
- she stands up to him⋆	1,706	74.56	74.30	74.82	0.802
- she refuses to have sex with him⋆	1,706	46.66	47.18	46.14	0.665
- she burns the meal⋆	1,706	21.10	21.13	21.08	0.980
In the last six months, did your husband: (%)					
- refuse to give you enough money for HH expenses⋆	1,706	18.58	18.66	18.50	0.932
- take money you earned on your own⋆	1,706	10.14	10.92	9.37	0.290
- try to keep you from seeing your friends or family⋆	1,706	10.73	10.33	11.12	0.596
- was jealous or angry if you had talked to other men⋆	1,706	12.43	13.26	11.59	0.296
- accuse you of being unfaithful⋆	1,706	6.27	6.69	5.85	0.477
- say something to humiliate you in the presence of others⋆	1,706	7.50	7.75	7.26	0.703
- threaten to hurt you or someone close to you⋆	1,706	4.63	4.81	4.45	0.722
Feels able to take contraceptives behind husband's back (%)⋄	1,683	15.39	14.42	16.35	0.273
Self-reported intimate partner violence (%)⋆	1,706	5.39	5.99	4.80	0.279
Husband's characteristics					
Age (in years)	1,161	36.92	36.89	36.95	0.904
Went to school (%)⋆	1,706	36.34	35.21	37.47	0.332
Number of wives⋆	1,706	1.32	1.33	1.32	0.710
Husband consumes alcohol (%)⋆	1,706	33.65	34.04	33.26	0.733
Family planning and contraception					
Wants another child (%)⋆	1,706	86.23	86.27	86.18	0.959
Number of desired children⋄	1,550	5.67	5.68	5.67	0.931
Do not know her husbands' number of desired children⋆	1,706	81.36	80.05	82.67	0.164
Last pregnancy was planned⋄	1,645	51.67	51.33	52.01	0.782
Use currently contraceptive methods (%)⋆	1,706	37.28	37.21	37.35	0.950
Husband alone makes decisions about contraception (%)⋆	1,706	40.97	39.91	42.04	0.371
Husband alone decides on the number of children to have (%)⋆	1,706	43.12	48.47	49.77	0.594
Husband does not approve contraception (%)	1,706	14.77	15.02	14.52	0.770
**Test of joint significance**					
considering the variables indicated by ⋆:		$F(35,1670)=0.44$, p-value=0.998			
considering the variables indicated by ⋆ and ⋄:		$F(39,1429)=0.52$, p-value=0.993			

Notes: N stands for number of observations. Differences in the number of observations is due to missing information.
† Reports the p-value of the difference of means between group 1 and group 2.
Variations in the number of observations is due to missing information.

Table A3: Checking floor, ceiling and design effects for the two list experiments

Estimated proportions	Source	Number of reported items (y)						Sum
		N	0	1	2	3	4	
Senegal								
**List A**								
Row 1	Treatment list	248	0	0.072	0.476	0.400	0.052	1
Row 2	$Pr(Y_i \leq y \| T_i = 1)$		0	0.072	0.548	0.948	1	
Row 3	Control list	247	0.024	0.405	0.486	0.085	-	1
Row 4	$Pr(Y_i \leq y \| T_i = 0)$		0.024	0.429	0.915	1	-	
Row 5	Row 4 - Row 2 (> 0)		0.024	0.357	0.367	0.052	-	0.800
Row 6	Row 2 - Row 4 ($y-1$) (> 0)		-	0.049	0.120	0.033	0	
**List B**								
Row 1	Treatment list	247	0.004	0.032	0.340	0.527	0.097	1
Row 2	$Pr(Y_i \leq y \| T_i = 1)$		0.004	0.036	0.376	0.903	1	
Row 3	Control list	248	0.024	0.161	0.718	0.097	-	1
Row 4	$Pr(Y_i \leq y \| T_i = 0)$		0.024	0.185	0.903	1	-	
Row 5	Row 4 - Row 2 (> 0)		0.020	0.149	0.527	0.097	-	0.793
Row 6	Row 2 - Row 4 ($y-1$) (> 0)		-	0.012	0.191	0	0	
Burkina Faso								
**List A**								
Row 1	Treatment list	852	0.043	0.371	0.446	0.121	0.019	1
Row 2	$Pr(Y_i \leq y \| T_i = 1)$		0.043	0.414	0.860	0.981	1	
Row 3	Control list	854	0.049	0.462	0.442	0.047	-	1
Row 4	$Pr(Y_i \leq y \| T_i = 0)$		0.049	0.511	0.953	1	-	
Row 5	Row 4 - Row 2 (> 0)		0.006	0.097	0.093	0.019	-	0.215
Row 6	Row 2 - Row 4 ($y-1$) (> 0)		-	0.365	0.349	0.028	0	
**List B**								
Row 1	Treatment list	854	0.034	0.393	0.428	0.125	0.020	1
Row 2	$Pr(Y_i \leq y \| T_i = 1)$		0.034	0.427	0.855	0.980	1	
Row 3	Control list	852	0.036	0.519	0.411	0.034	-	1
Row 4	$Pr(Y_i \leq y \| T_i = 0)$		0.036	0.555	0.966	1	-	
Row 5	Row 4 - Row 2 (> 0)		0.002	0.128	0.111	0.020	-	0.261
Row 6	Row 2 - Row 4 ($y-1$) (> 0)		-	0.391	0.300	0.014	0	

Notes: N stands for the number of observations. The sum of the difference between Row 4 and Row 2 gives the difference-in-means estimator (cf. results presented in Table 1).

A2 Additional tables and figures

Mbao site Pikine site

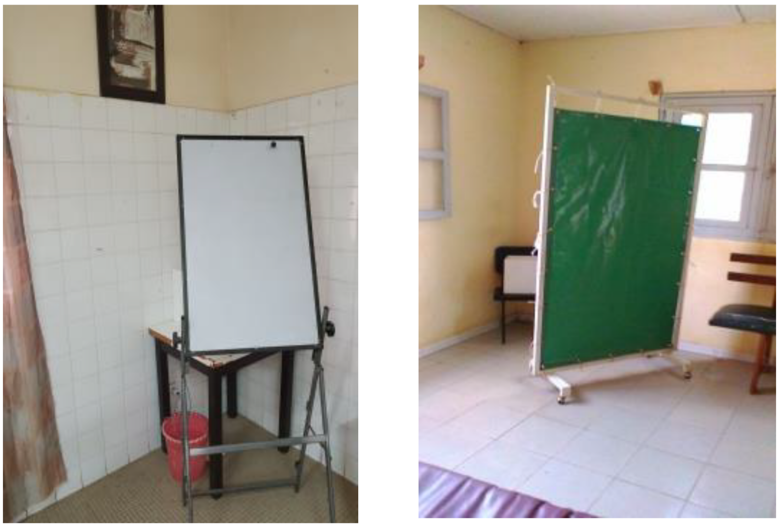

Figure A1: Polling box settings

Table A4: Condom use estimated via different methodologies

	Condom use (%)		
**Health facility**	Self-reported by FSWs †	Polling box ‡	Double list experiment ∓
Pikine	96.99	85.16	72.81
Mbao	99.26	90.15	91.31
Rufisque	91.96	85.47	70.05
Sebikotane	98.28	87.50	82.20
Total	96.77	88.14	79.60

† Obs: Pikine ($N = 133$); Mbao ($N = 135$); Rufisque ($N = 112$); Sebikotane ($N = 116$); Total ($N = 496$).
‡ Obs: Pikine ($N = 155$); Mbao ($N = 132$); Rufisque ($N = 117$); Sebikotane ($N = 112$); Total ($N = 516$).
∓ Obs: Pikine ($N = 133$); Mbao ($N = 135$); Rufisque ($N = 111$); Sebikotane ($N = 116$); Total ($N = 496$).

A3 Bias-variance tradeoff

We use the same notations as in **?**: $Y_i(1)$ is the number of statements an individual i would give if in the treated group and $Y_i(0)$ the number of statements this individual would give if in the control group. p_i is the observed answer to the direct question while p_i^* is the latent behaviour. These variables take the value 1 if the respondent declare and actually adopt the sensitive behaviour respectively. $\pi^* = E(p_i^*)$ is the true sensitive behaviour prevalence rate. $W_i = p_i^* - p_i$ is the difference for one individual between the true behaviour and the declared one. It takes value 0 if the individual tells the truth and value 1 if she lies. $E(W_i) = B$, B refers to the bias.

Following **?**, we considered the mean squared-error of the list experiment (MSE_L) formula presented by **?**:

$$MSE_L = \frac{1}{N-1}\{\frac{mVar[Y_i(0)]}{N-m} + \frac{(N-m)Var[Y_i(1)]}{m} + 2Cov[Y_i(0), Y_i(1)]\}$$

In the classic list experiment design that we followed, half of the women are allocated to the treated group (i.e. list with the sensitive item) and the other half to the control group. Thus, $m = \frac{N}{2}$. Furthermore, the no design effect and no "liars" assumptions imply that $Y_i(1) = Y_i(0) + p_i^*$ and that $Y_i(0) \perp p_i^*$, the mean squared-error of the list experiment can thus be simplified as follows:

$$MSE_L = \frac{4Var[Y_i(0)] + Var(p_i^*)}{N-1}$$

As for the mean squared-error of the direct question (MSE_D), it is equal to:

$$MSE_D = \frac{Var(p_i)}{N} + B^2$$

We are interested in studying the values of N and B for which $MSE_L < MSE_D$. This is the case when:

$$\frac{4Var[Y_i(0)] + Var(p_i^*)}{N-1} < \frac{Var(p_i)}{N} + B^2 \tag{1}$$

Given the definition of W_i, the fact that W_i and p_i^* are two Bernouilli variables and $W_i = 1$ implies $p_i^* = 1$, $Var(p_i)$ can be expressed as a function of B and π^*:

$$Var(p_i) = -B^2 + (2\pi^* - 1)B + \pi^*(1-\pi^*)$$

We replace $Var(p_i)$ and $Var(p_i^*)$ in equation (1):

$$\frac{4Var[Y_i(0)] + \pi^*(1-\pi^*)}{N-1} < \frac{-B^2 + (2\pi^* - 1)B + \pi^*(1-\pi^*)}{N} + B^2$$

We multiply both sides by $N(N-1)$ and solve the equality:

$$B^2(N-1)^2 + (2\pi^* - 1)B(N-1) - 4Var[Y_i(0)](N-1) - \pi^*(1-\pi^*) - 4Var[Y_i(0)] = 0$$

Let's note $(x, y) = (N-1, B)$ and $C = \pi^*(1-\pi^*) + 4Var[Y_i(0)]$, the previous expression simplified as follows:

$$y^2x^2 + (2\pi^* - 1)yx - 4Var[Y_i(0)]x - C = 0 \tag{2}$$

We can use equation (2) to compute the sample size required (N), according to the bias-variance trade-off criteria, to use the list experiment given the bias (B) and the variance in the answers provided by the control group ($Var[Y_i(0)]$) observed in each of our two studies.
